# Factorial Structure and Psychometric Properties of the Spanish Version of the Pittsburgh Sleep Quality Index in Non-Professional Caregivers

**DOI:** 10.3390/healthcare11010067

**Published:** 2022-12-26

**Authors:** Patricia Otero, Miguel A. Simón, Ana M. Bueno, Vanessa Blanco, Fernando L. Vázquez

**Affiliations:** 1 Department of Psychology, University of A Coruña, 15071 A Coruña, Spain; 2 Department of Evolutionary and Educational Psychology, University of Santiago de Compostela, 15782 Santiago de Compostela, Spain; 3 Department of Clinical Psychology and Psychobiology, University of Santiago de Compostela, 15782 Santiago de Compostela, Spain

**Keywords:** PSQI, sleep, caregivers, reliability, internal consistency, validity, factor analysis, Spanish

## Abstract

Although sleep issues are among the symptoms commonly experienced by the non-professional caregiver population, and the Pittsburgh Sleep Quality Index (PSQI) is the most widely used instrument for the assessment of sleep quality, this has not been validated specifically for this population. The objective of this study was to analyze the factorial structure and psychometric properties of the Spanish version of the PSQI in a sample of Spanish non-professional caregivers. Trained clinical psychologists assessed sleep quality using the PSQI, as well as caregiver burden and psychological distress in 201 non-professional caregivers (87.1% female, *M*_age_ = 56.2 years). The internal consistency of the PSQI was 0.75. The two-factor model (Sleep quality and Disturbances) had an acceptable fit to the data, was found to be superior to the one-factor model, and more parsimonious than the three-factor model. There was a significant correlation between the PSQI and caregiver burden, as well as between the PSQI and psychological distress (*p* < 0.001 in all cases). A total score ≥ 9 allowed the identification of caregivers with possible anxiety and depression disorders (sensitivity 70.5%, specificity 71.9%). The results show that the PSQI is a reliable and valid instrument for the assessment of sleep quality in caregivers.

## 1. Introduction

Being a non-professional caregiver who provides care for a dependent loved one is a challenging and stressful task that demands enormous dedication and continuous effort. Previous research has shown that playing this role can negatively impact the caregiver’s emotional and physical well-being [1], so caregivers may develop depression, anxiety, and burden [2,3] or experience back injuries, hypertension, or headaches [4]. In addition, sleep disturbances are common among non-professional caregivers, often linked to the sleep disturbances or nighttime behaviors of the care recipient [5]. Previous studies have reported that 41.0% of caregivers experience insomnia [6] and 76.1% suffer from poor sleep quality [7]. Sleep quality refers to a complex construct that includes quantitative aspects of sleep such as sleep duration, sleep latency, and sleep efficiency, as well as subjective aspects, such as restfulness of sleep or daytime dysfunction [8]. Specifically, it was found that caregivers experienced a decrease in total sleep time, fragmented sleep due to frequent nighttime awakenings, and increased daytime sleepiness and fatigue [9,10]. 

Sleep problems lead to adverse health consequences and a poor quality of life. They are linked to the occurrence of cognitive dysfunctions, such as attention deficit, impaired cognitive performance, and emotional dysregulation, an increase in occupational and motor vehicle accidents, and poor general health [11,12]. Therefore, poor sleep quality can have a negative impact not only on caregivers’ own health, daily functioning, and well-being, but also on the quality of care they are able to provide to their dependent loved ones. In fact, one of the most common reasons for the institutionalization of the care recipient are their sleep disturbances, often because this disrupts the caregiver’s sleep [13].

A sleep quality assessment is an essential first step to plan the most appropriate intervention for the caregivers’ sleep problems. In this context, the Pittsburgh Sleep Quality Index (PSQI) is the most validated and widely used instrument for the assessment of overall sleep quality [14,15], and is considered a suitable assessment tool for research [16]. The instrument has been shown to offer good overall reliability and validity, and it is widely used as a screening tool to identify sleep problems. A systematic review and meta-analysis of 37 studies [15] found that the PSQI had good internal consistency (the Cronbach’s α of the studies reviewed ranged from 0.70 to 0.83). The tool showed a moderate positive association with theoretically related variables, such as depression and anxiety, and a weak association with unrelated variables, such as perceived social support and anger. 

To date, the PSQI has been translated and validated in more than 40 countries, including Brazil [17], Colombia [18], Nigeria [19], China [20], Japan [21], Portugal [22], Italy [23], Germany [24], and Spain [25]. It has also been validated in different populations, such as adolescents [26], older adults [27,28], kidney transplant recipients [29], fibromyalgia patients [30], multiple sclerosis patients [31], breast cancer patients [32], patients with chronic pain [33], and women with post-traumatic stress disorder [34], among others. However, to the best of our knowledge, its psychometric properties have never been validated in the non-professional caregiver population.

On the other hand, the factor structure of the PSQI has generated a great deal of debate and requires further research [14,15]. The original version of the PSQI assumed that a global score representing overall sleep quality was sufficient to capture the multifaceted nature of the sleep problems that the instrument identified. However, since the first published research on the dimensionality of the instrument [27], numerous subsequent studies have reported various factor structures that support the existence of one factor (e.g., [26,32,33,35,36]), two factors (e.g., [28,30,31,37,38]), and three factors (e.g., [27,29,34,39,40]). Thus, there is no consensus on the factor structure, nor is there any study that has analyzed the factor structure of the PSQI in the population of non-professional caregivers. 

The objective of this study was to analyze the factorial structure and psychometric properties of the Spanish version of the PSQI in a sample of Spanish non-professional caregivers. 

## 2. Materials and Methods

### 2.1. Participants

This study is part of a larger research project on sleep and burden of non-professional caregivers using unique cohort participants. A cross-sectional study was conducted using a simple random sample of caregivers listed in the official registry of caregivers compiled by the Ministry of Labor and Welfare of the regional government of Galicia (Spain), a region in northwestern Spain with 2,732,347 inhabitants. To this end, we signed an agreement with the Ministry of Labor and Welfare to facilitate contact with the caregivers. 

To participate in this study, each participant had to be a non-professional caregiver living with a care recipient whose dependence was officially recognized by the Ministry of Labor and Welfare and had to provide their informed consent. Participants were excluded if they had any communication difficulties (e.g., unable to read or write), any condition that could interfere with their participation (e.g., significant cognitive impairment, severe visual impairment), or if they had received psychological or pharmacological treatment in the previous two months.

The response rate was 95.7%. Of the 210 caregivers contacted to participate in the study, 9 declined to participate, resulting in a final sample of 201 caregivers. 

The study was conducted in accordance with the latest version of the Declaration of Helsinki and was approved by the Bioethics Committee of the University of Santiago de Compostela (Code number 07092016). The participation was voluntary, and participants received no financial compensation or any other incentive. Additionally, all participants gave their informed consent to participate in the study.

### 2.2. Measures

An ad hoc questionnaire was used to record sociodemographic variables (sex, age, marital status, monthly family income) and the caregiving situation (relationship to the caregiver, sex, age, and condition of the dependent person, years spent as a caregiver, and daily hours dedicated to care).

The Pittsburgh Sleep Quality Index (PSQI) [8], Spanish version of Royuela and Macías [25], was utilized to assess sleep quality. This instrument includes a total of 19 items, divided into 7 components: (1) Subjective sleep quality, (2) Sleep latency, (3) Sleep duration, (4) Habitual sleep efficiency, (5) Sleep disturbances, (6) Use of sleep medication, and (7) Daytime dysfunction. The PSQI total score ranges from 0 to 21, with higher scores indicating poorer sleep quality, and an overall score of more than 5 indicating a “poor” sleeper. The Spanish version of the PSQI has adequate internal consistency (Cronbach’s α = 0.67–0.81 in two samples of university students and a clinical sample formed by psychiatric and primary care patients). 

The Caregiver Burden Inventory (CBI) [41], Spanish version validated by Vázquez et al. [42], was used to assess the caregiver burden. It is composed of 24 items with a Likert-type response scale from 0 to 4. Therefore, its overall score ranges from 0 to 96, with higher scores indicative of a greater burden. The Spanish version has adequate internal consistency (Cronbach’s α = 0.89).

The General Health Questionnaire (GHQ-12) [43], Spanish version of Rocha et al. [44], was used to assess psychological distress. This instrument consists of 12 items, with global scores that can range from 0 to 12, with higher scores indicating greater emotional distress. Its internal consistency is 0.86 for people under 65 years of age and 0.90 for people 65 years of age and older. The cutoff point of 2/3 allows the detection of the possible presence of psychopathology (i.e., possible cases of anxiety and depression disorders), with a sensitivity of 76% and a specificity of 80% [45].

### 2.3. Procedure

We started by compiling a list of all caregivers of the database (*n* = 18,410) of the Ministry of Labor and Welfare of Galicia, and then assigned a sequential number to each person (1, 2, 3, ..., 18,410). After calculating the desired sample size (*n* = 210), we selected participants randomly using a random number generator to generate 210 random numbers between 1 and 18,410.

Once the sample of study participants was drawn, the caregivers were contacted by letter or telephone call, the characteristics of the study were explained to them, and they were invited to participate.

Three previously trained clinical psychologists helped the participants self-administer the questionnaire on sociodemographic and caregiving characteristics, the PSQI, the CBI, and the GHQ-12 in-person at public community centers near the caregivers’ homes, from February to October 2018. Each assessment took approximately 45 min to complete.

To minimize dropouts, we followed the strategies recommended by Hulley et al. [46], such as excluding participants who were likely to be lost, making the study presentation attractive, treating participants with kindness, affection, and respect, and helping them understand the research question so that they would want to participate in making the study successful.

### 2.4. Data Analysis

Analyses were performed using the statistical package SPSS for Windows (version 26, IBM, Armonk, NY, USA) and SPSS Amos Graphics (version 26, IBM, Chicago, IL, USA).

We calculated the frequency, percentages, mean, and standard deviation of the sociodemographic and care variables, and the PSQI total score. In addition, Student’s *t*-test, ANOVA, and Pearson correlations were used to analyze differences in PSQI total scores as a function of sociodemographic characteristics and the caregiving situation.

Cronbach’s α was calculated to analyze the internal consistency of the PSQI. We calculated the Pearson correlations between the items and between the score of each item and the total corrected score (i.e., the total score without considering that item). 

To explore the underlying factors of the PSQI in the population of caregivers, we performed a factor analysis. First, the Kaiser–Meyer–Olkin (KMO) measure and Bartlett’s Test of Sphericity were performed to determine the suitability of this sample for factor analysis. The KMO indicates the proportion of variance in the variables that might be caused by underlying factors. High values (closer to 1.0) are considered ideal, while values less than 0.5 are unacceptable for a satisfactory factor analysis. The Bartlett’s Test of Sphericity was used to test the null hypothesis that the correlation matrix is an identity matrix, which would indicate that the variables are unrelated. A significant statistical test shows that the correlation matrix is not an identity matrix (rejection of the null hypothesis), and therefore is appropriate for factor analysis. Following Manzar et al.’s [14] recommendation, a cross-validation approach combining the exploratory and confirmatory factor analysis was undertaken to assess the factor structure of the PSQI. The sample was randomly split into two independent subsamples to perform an exploratory factorial analysis (EFA) and confirmatory factor analysis (CFA).

EFA was performed on the first randomly assigned sample (*n* = 100) using principal components’ extraction and orthogonal varimax rotation. To determine the number of factors retained, we used both Cattell’s scree test and the Kaiser criterion. According to the scree test, the number of factors to retain was established through visual inspection of the shape of the curve by detecting the point at which the eigenvalue curve changes drastically. According to the Kaiser criterion, the number of factors retained was equal to the number of factors with an eigenvalue greater than one. Factor loadings were evaluated following criteria from Comrey and Lee [47]: 0.71 or greater are excellent loadings, 0.63 to 0.70 are very good, 0.55 to 0.62 are good, 0.45 to 0.54 are fair, 0.32 to 0.44 are poor, and any values lower than 0.32 are discarded.

CFA was performed on the second randomly assigned sample (*n* = 101) using the maximum likelihood method to verify the factorial structure of the questionnaire. In this analysis, we tested the model identified through the EFA, the single-factor structure proposed in the original version by Buysse et al. [8], and the three-factor structure proposed by Cole et al. [27]. The goodness of fit was assessed using the following indices: (a) the chi-square test, which assesses overall fit and the discrepancy between the sample and fitted covariance matrices (non-significant values indicate good model fit), (b) the values of the parsimony-adjusted index Root Mean Square Error of Approximation (RMSEA) ≤ 0.06, (c) the Goodness of Fit Index (GFI) > 0.90, which shows the proportion of variance accounted for by the estimated population covariance, (d) the Adjusted Goodness-of-Fit Index (AGFI) > 0.90 (the adjusted form of the GFI), (e) the Normalized Fit Index (NFI) close to 0.95, which indicates that the model of interest improves the fit, (f) the Comparative Fit Index (CFI, a revised form of NFI) close to 0.95, and (g) lower values of the Expected Cross-Validation Index (ECVI), which measures the predicted future of a model using simple transformation of chi-square) [48,49]. 

To examine the criterion validity of the PSQI, we used Pearson’s correlation between the PSQI and the CBI, and between the PSQI and the GHQ-12, Student’s *t*-test for independent samples, and a discriminant classification analysis, which allow the classification of the possible cases of psychopathology (anxiety and depression disorders). A receiver’s operating characteristics (ROC) curve analysis was performed to determine the optimal cutoff point to discriminate possible psychopathology (anxiety and depression). Sensitivity (i.e., the probability that a test will indicate “disorder” among those with the disorder), specificity (i.e., the fraction of those without the disorder who will have a negative test), positive predictive value (i.e., the proportion of people with a positive test result who actually have the disorder), and negative predictive value (i.e., the proportion of those with a negative result who do not have the disorder) were calculated. 

## 3. Results

### 3.1. Sample Profile and Sleep Quality

Table 1 shows the sociodemographic and caregiving status characteristics of the full sample and the two subsamples. In the full sample, 87.1% were female, with a mean age of 56.2 years (SD = 10.1). In addition, 79.6% had a partner and 55.7% had a monthly family income between 1000 and 1999 Euros. Regarding the caregiving situation, 43.8% took care of their father or mother. The care recipient was female in 55.7% of cases, with a mean age of 71.6 years (SD = 21.5), and 54.6% of care recipients had a physical disability. Caregivers had spent an average of 14.5 years providing care (SD = 11.7), devoting 16.2 hours per day (SD = 5.3) to caregiving tasks. There were no significant differences between the two subsamples for any of the sociodemographic variables or caregiving variables.

The overall mean score on the PSQI was 9.0 (SD = 4.3). Using the recommended cutoff point > 5 for the PSQI global score, 76.1% of participants had poor sleep quality. Sleep quality was significantly related to the number of hours spent caregiving (*r* = 0.213, *p* = 0.002) and was significantly worse for caregivers caring for a family member with an intellectual disability (*p* < 0.001) or cognitive impairment (*p* < 0.001) compared to those caring for a family member with a physical disability. There were no significant differences in any other sociodemographic or caregiving status variables.

### 3.2. Reliability

Table 2 shows means, standard deviations, score frequency, and corrected item-total correlation for each component of the PSQI. The mean scores for the seven components ranged from 0.7 (SD = 1.1) for Use of sleep medication to 1.7 (SD = 1.1) for Sleep latency. Of the components, 26.5% had scores of 0, 32.5% had scores of 1, 27.1% had scores of 2, and 13.9% had scores of 3.

The total PSQI showed an internal consistency of 0.75. The corrected item-total correlation coefficients were all significant (*p* < 0.001) and ranged from 0.24 for Use of sleep medication to 0.66 for Habitual sleep efficiency. The mean of the inter-item correlation coefficient was 0.317, with a minimum value of 0.115 and a maximum of 0.689.

### 3.3. Validity

#### 3.3.1. Factor Structure

##### Exploratory Factor Analysis

The KMO = 0.821 and Bartlett’s Test of Sphericity (χ^2^_(21)_ = 189.932, *p* < 0.001) verified that exploratory factor analysis is applicable in this sample. When the principal component factor analysis was conducted, the results revealed that in the sample of caregivers, the PSQI consisted of two factors, which explained 59.5% of variance. The first factor, called *Sleep quality*, explains 36.6% of the variance and includes Sleep efficiency, Sleep duration, Sleep latency, and Subjective sleep quality. The second factor, called *Disturbances*, explains 23.0% of the variance and includes Daytime dysfunction, Sleep disturbances, and Use of sleep medication. As Table 3 shows, four of the components had excellent loadings (0.784–0.858), two had very good loadings (0.676–0.695), and one had a good loading (0.555).

##### Confirmatory Factor Analysis

We compared our two-factor model with the one-factor model of Buysse et al. [8] and the three-factor model suggested by Cole et al. [27] (see Table 4). The model with only one factor had a significant chi-square, RMSEA = 0.112, GFI = 0.913, AGFI = 0.826, CFI = 0.900, NFI = 0.839, and ECVI = 0.594, indicating a poor fit. Factor loadings ranged from 0.23 to 0.83 and were very low for Sleep disturbances (0.34), Use of sleep medication (0.23), and Daytime dysfunction (0.24). 

**Figure 1:**
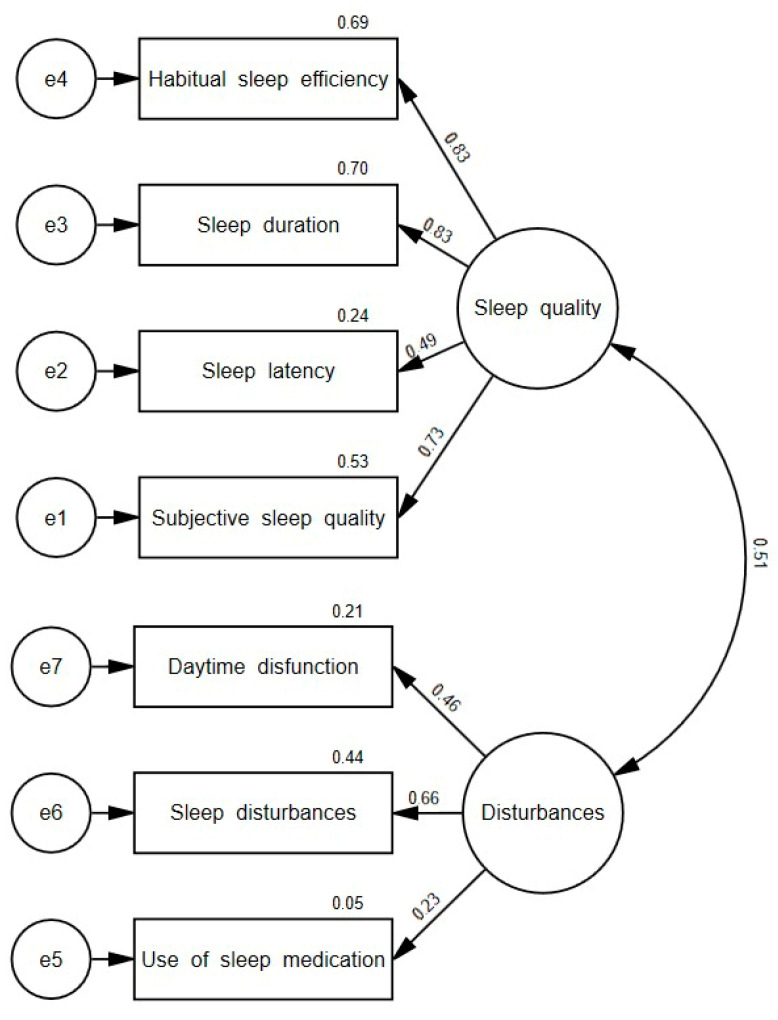
Factor loadings for the two-factor model.

The two-factor model identified through our EFA had a significant chi-square, RMSEA = 0.097, GFI = 0.930, AGFI = 0.850, CFI = 0.929, NFI = 0.870, and ECVI = 0.553, indicating a sufficient, albeit moderate, fit to the data. Factor loadings ranged from 0.23 to 0.83, most being satisfactory (0.66–0.83), with fair values for Daytime dysfunction (0.46) and Sleep latency (0.49) and a low value for Use of sleep medication (0.23) (see Figure 1).

The three-factor model obtained a non-significant chi-square, RMSEA = 0.000, GFI = 0.971, AGFI = 0.925, CFI = 1.000, NFI = 0.944, and ECVI = 0.449, indicating a good fit. The factor loadings were satisfactory (0.51–0.85), with the exception of the Use of sleep medication component, which showed a loading of 0.22.

#### 3.3.2. Relationship between the PSQI and Other Questionnaires

A significant positive correlation was found between the total sleep quality score on the PSQI and the total caregiver burden score on the CBI (*r* = 0.494, *p* < 0.001), as well as between burden and the factors of Sleep quality (*r* = 0.404, *p* < 0.001) and Disturbances (*r* = 0.457, *p* < 0.001). 

There was also a significant positive correlation between the total sleep quality score on the PSQI and the total psychological distress score on the GHQ-12 (*r* = 0.626, *p* < 0.001), and between psychological distress and the factors of Sleep quality (*r* = 0.501, *p* < 0.001) and Disturbances (*r* = 0.599, *p* < 0.001). 

**Figure 2:**
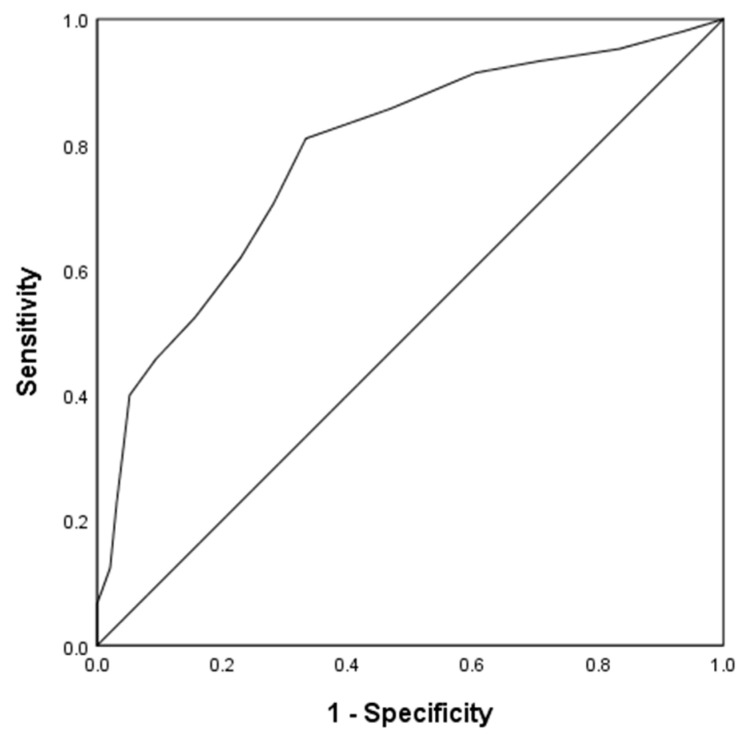
Receiver operating characteristics (ROC) curve.

In addition, the Student’s *t*-test indicated that caregivers who were poor sleepers experienced greater psychological distress, *t* _(116.10)_ = −8.24, *p* < 0.001. Using discriminant classification analysis, the Wilks’ lambda was 0.76, χ^2^_(1,n=201)_ = 54.17, *p* < 0.001, and the canonical correlation was 0.489, correctly classifying 71.1% of cases. The area under the ROC curve was 0.78 (95% CI 0.72–0.85) (Figure 2). Those who scored 9 or more on the PSQI not only had poor sleep quality but were also likely to show psychopathology such as anxiety and depression disorders (with a sensitivity of 70.5%, a specificity of 71.9%, a positive predictive value of 73.3%, and a negative predictive value of 69.0%).

## 4. Discussion

The aim of this study was to analyze the factorial structure and psychometric properties of the Spanish version of the PSQI in a sample of Spanish non-professional caregivers. The mean PSQI score obtained in this sample was 9.0. This is higher than that found in clinical samples of patients with chronic pain (M = 7.67) [33], multiple sclerosis (M = 7.36) [31], or breast cancer (M = 7.59) [32] with the PSQI, and is also higher than that found in non-clinical samples of older persons (M = 4.98 [27], M = 5.98 [39]), centenarians (M = 8.44) [28], and adolescents (M = 7.36) [25] through the same instrument. However, the mean score was lower than that found in samples of patients with post-traumatic stress disorder (M = 11.26) [34] and patients with fibromyalgia (M = 13.22) [30], also through the PSQI. Sleep quality was significantly worse for those caregivers who devoted more hours per day to caregiving and who cared for a family member with an intellectual disability or mental disorder (*p <* 0.001) or those with cognitive impairment (*p <* 0.001), compared to those who cared for a family member with a physical disability. One possible explanation is that providing care for people with mental disorders or cognitive impairment is more complex and demanding and may require added nighttime care due to disruptive nighttime behaviors, which increases the number of hours spent giving care and decreases the caregiver’s quality of sleep. This finding is consistent with previous studies using the PSQI [7,50]. Therefore, these variables are particularly important when addressing the needs of caregiver populations, both in clinical practice and in research.

The overall internal consistency of the PSQI was acceptable (α = 0.75). Although Cronbach’s alpha was lower than the 0.83 found in the original version of the instrument [8], these values meet the criteria recommended by Streiner et al. [51] for the health measurement scales and are consistent with the values reported in the Spanish version of the instrument (α = 0.67–0.81 in two samples of students and one sample of a clinical population) [25], and those reported in the PSQI for samples of chronic pain patients, pregnant women (α = 0.74) [33,52], and adolescents (α = 0.73) [26]. 

The EFA identified two factors in the PSQI: Sleep quality and Disturbances. This two-factor model is consistent with numerous studies (e.g., [28,30,31,37,38]) and is the factor structure most frequently found in the scientific literature [14]. Specifically, the factor composition of the PSQI model found was similar to that found in the studies by Hita-Contreras et al. [30] and Zhang et al. [28], but different from the two-factor model broken down into Sleep efficiency and Perceived sleep quality found in other studies (e.g., [31,38]). The CFA showed that the two-factor model, together with the three-factor model proposed by Cole et al. [27], were favored statistically over the single-factor model proposed by Buysse et al. [8]. These results are consistent with other studies about PSQI factorial structure (e.g., [38,53]) and suggest that a single factor does not capture the multidimensional nature of sleep quality. Given that the recommended practice for factor analysis gives preference to more parsimonious models [54], the two-factor factor model may be considered the most appropriate for the caregiver population. 

A significant positive correlation was found between lower sleep quality and higher caregiver burden. This could be because caregivers’ sleep problems have an impact on the caregiving tasks they perform, as a result of lower concentration, more mistakes, and reduced patience, which increases caregivers’ perception of burden. These results are consistent with those found in previous studies of caregiver populations using the PSQI [7,55] and an insomnia diagnostic interview [6]. On the other hand, a significant positive correlation was found between lower sleep quality and greater psychological distress, indicating a relationship between PSQI and possible mental health problems (anxiety and depression) in caregivers. Indeed, there is evidence showing that sleep problems in adults (assessed through an open-ended questionnaire regarding sleep habits, occurrence and frequency of trouble either falling and/or staying asleep) are a risk factor for the occurrence of affective symptoms (such as emotional dysregulation, irritability, anxiety, and depression), cognitive symptoms (such as lack of concentration or intrusive thoughts), and somatic symptoms (such as headache, muscle tension, fatigue, and body temperature dysregulation) [56]. Additionally, this study found that a total score ≥ 9 on the PSQI constitutes an optimal cutoff point that discriminates between caregivers with possible psychopathologies. 

Despite the interesting findings of this study, we must consider some limitations. The self-report nature of the instruments could generate response bias and artificially increase correlations between variables [57]. Despite being a recognized screening instrument, the use of the GHQ-12 to assess possible psychopathology (specifically anxiety and depression disorders) should be interpreted with caution because it does not establish clinical diagnoses. In this sample, all caregivers slept in the same house as their care recipient, and we do not have information about how many caregivers slept in the same room as the person cared for. Future studies could differentiate between caregivers that sleep in the same house or room as their care recipient. Given the nature of the sample, the findings may not be generalizable to other populations. Finally, due to the type of design, test–retest reliability could not be performed.

It should be noted that this study has important implications for research and clinical practice. This is the first study that provides information on the factorial structure and psychometric properties of the PSQI in a Spanish non-professional caregiver population, showing that it can be used by researchers and clinicians interested in assessing sleep quality in this population. Health professionals should be attentive to detect sleep problems, which imply enormous economic costs in medical consultations, use of health services, consumption of medicines, loss of labor productivity, increased probability of accidents [58], and institutional care for the care recipient [13]. The CFA revealed that a two-factor model provided an acceptable fit to the data and was found to be superior to the one-factor model and more parsimonious than the three-factor model. If these factors are not considered, researchers and clinicians may miss specific aspects of sleep impairment that may only reside in one of the factors. This is important because practitioners need to know as much as possible about the type and nature of sleep problems to guide the treatment approach [59]. In addition, this study provides cutoff points that are capable of identifying caregivers with possible psychopathologies. This is especially useful for identifying caregivers in urgent need of psychological or psychiatric care or at risk of institutionalizing their care recipient.

## 5. Conclusions

In conclusion, this study provided evidence of the psychometric properties and the two-factor structure of the PSQI in a sample of non-professional caregivers. The results showed that the PSQI is a reliable and valid instrument for assessing sleep quality in the caregiver population.

## Figures and Tables

**Table 1:** Participants’ sociodemographic and care characteristics.

Variables	*n* = 201 (%)	Subsample 1 *n* = 100 (%)	Subsample 2 *n* = 101 (%)	Comparison between Subsamples	
Sex					
Male	26 (12.9)	14 (14.0)	12 (11.9)	χ^2^_(1,N=201)_ = 0.20, *p* = 0.654	
Female	175 (87.1)	86 (86.0)	89 (88.1)		
Age					
M (SD)	56.2 (10.1)	56.9 (10.1)	55.4 (10.0)	*t*_(199)_ = 1.10, *p* = 0.272	
Marital status					
Single	41 (20.4)	21 (21.0)	20 (19.8)	χ^2^_(1,N=201)_ = 0.44, *p* = 0.833	
Partnered	160 (79.6)	79 (79.0)	81 (80.2)		
Monthly family income					
<999 Euros	71 (35.3)	41 (41.0)	30 (29.7)	F de Fisher, *p* = 0.061	
Between 1000 and 1999 Euros	112 (55.7)	55 (55.0)	57 (56.4)		
>2000 Euros	18 (9.0)	4 (4.0)	14 (13.9)		
Relationship with the person cared for					
Parent	88 (43.8)	38 (38.0)	50 (49.5)	χ^2^_(2,N=201)_ = 2.87, *p* = 0.238	
Daughter/son	42 (20.9)	22 (22.0)	20 (19.8)		
Other relatives	71 (35.3)	40 (40.0)	31 (30.7)		
Sex of the person cared for					
Male	89 (44.3)	42 (42.0)	47 (46.5)	χ^2^_(1,N=201)_ = 1.50, *p* = 0.472	
Female	112 (55.7)	58 (58.0)	54 (53.5)		
Age of the person cared for					
M (SD)	71.6 (21.5)	70.1 (19.8)	72.9 (23.1)	*t*_(199)_ = −0.94, *p* = 0.348	
Condition of the person cared for					
Intellectual disability or mental disorder	35 (17.5)	22 (22.0)	13 (12.8)	χ^2^_(2,N=201)_ = 5.02, *p* = 0.081	
Physical disability	110 (54.6)	47 (47.0)	63 (62.4)		
Cognitive impairment	56 (27.9)	31 (31.0)	25 (24.8)		
Time dedicated to care (years)					
M (SD)	14.5 (11.7)	15.3 (12.4)	13.7 (10.9)	*t*_(199)_ = 0.97, *p* = 0.332	
Daily hours dedicated to care					
M (SD)	16.2 (5.3)	16.5 (5.7)	16.0 (4.9)	*t*_(199)_ = 0.80, *p* = 0.426	

Note: M = mean; SD = standard deviation.

**Table 2:** Means, standard deviations, score frequency, and corrected item-total correlation for each component of the PSQI.

PSQI Component	M	SD	Score Frequency (%)				r^tot^
			0	1	2	3	
Subjective sleep quality	1.4	0.8	10.4	45.8	35.3	8.5	0.64
Sleep latency	1.7	1.1	21.4	23.9	22.4	32.3	0.52
Sleep duration	1.3	0.9	20.9	33.8	36.3	9.0	0.61
Habitual sleep efficiency	1.2	1.2	37.3	24.9	16.9	20.9	0.66
Sleep disturbances	1.4	0.6	3.0	55.2	37.8	4.0	0.42
Use of sleep medication	0.7	1.1	72.6	6.0	5.5	15.9	0.24
Daytime dysfunction	1.3	0.9	19.9	37.8	35.8	6.5	0.32
Total Cronbach’s α							0.75
Mean inter-item correlation coefficient							0.317

Note: PSQI = Pittsburgh Sleep Quality Index; M = mean; SD = standard deviation; r^tot^ = corrected item-total correlation.

**Table 3:** Exploratory factor analysis for PSQI.

PSQI Component	Factor 1 (*Sleep Quality*)	Factor 2 (*Disturbances*)
Habitual sleep efficiency	0.858	
Sleep duration	0.811	
Sleep latency	0.789	
Subjective sleep quality	0.676	
Daytime dysfunction		0.784
Sleep disturbances		0.695
Use of sleep medication		0.555
Variance explained (%)	36.6	23.0

Note: PSQI = Pittsburgh Sleep Quality Index. Principal components’ extraction and orthogonal varimax rotation.

**Table 4:** Confirmatory factor analysis for PSQI.

Model	Χ^2^ (df)	RMSEA	GFI	AGFI	CFI	NFI	ECVI
1 factor	31.420 (14) *	0.112	0.913	0.826	0.900	0.839	0.594
2 factors	25.321 (13) *	0.097	0.930	0.850	0.929	0.870	0.553
3 factors	10.935 (11)	0.000	0.971	0.925	1.000	0.944	0.449

Note: *Χ*^2^ (df) = chi-square test (degrees of freedom); RMSEA = Root Mean Square Error of Approximation; GFI = Goodness of Fit Index; AGFI = Adjusted Goodness-of-Fit Index; CFI = Comparative Fit Index; NFI = Normalized Fit Index; ECVI = Expected Cross-Validation Index. * *p* < 0.05.

## Data Availability

The data presented in this study are available upon request from the corresponding author. The data are not publicly available due to confidentiality issues.
